# Should colloid boluses be prioritized over crystalloid boluses for the management of dengue shock syndrome in the presence of ascites and pleural effusions?

**DOI:** 10.1186/1471-2334-11-52

**Published:** 2011-02-28

**Authors:** Ranjan Premaratna, Erandi Liyanaarachchi, Mindu Weerasinghe, H Janaka de Silva

**Affiliations:** 1 Department of Medicine, Faculty of Medicine, University of Kelaniya, Sri Lanka

## Abstract

**Background:**

Although the WHO guideline for the management of dengue fever considers the presence of ascites or pleural effusions in the diagnosis of DSS, it does not emphasize the importance of their presence when selecting fluids for resuscitation.

**Case presentation:**

We highlight three patients with DSS who received boluses of crystalloids on priority basis as recommended by WHO guidelines during resuscitation. All three patients had varying degrees of third space fluid loss (ascites and pleural effusions) at the time of development of DSS. Ascites and pleural effusions were detected in all 3 patients at the time of shock irrespective of whether iv fluids were given or not. All three patients had documented liver involvement at the time of shock evidenced by elevation of AST (4800 iu/L, 5000 iu/L and 1960 iu/L). One patient who had profound shock died 6 hours after admission with evidence of acute pulmonary oedema in the convalescence phase. All of them needed CPAP ventilator support and potent diuretics.

**Conclusions:**

We therefore feel that resuscitation of patients with DSS who already have third space fluid accumulation with crystalloid boluses on priority basis may contribute to recovery phase pulmonary oedema.

## Background

Dengue fever poses a major challenge to clinicians practicing in areas endemic for the infection. The presence of four serotypes, immunity related subsequent enhancement in severity, inadequate understanding of the pathophysiology of severe infection [1,2], absence of specific treatment, overcrowding in hospitals, inadequate laboratory facilities, delayed admissions due to patient ignorance and delayed diagnosis all contribute to the associated morbidity and mortality.

The diagnosis and management of severe dengue fever (DF), dengue shock syndrome (DSS) and dengue haemorrhagic fever (DHF) are based mainly on clinical and haematological parameters [3-5]. The WHO guidelines which were practiced until September 2009 [3,4] and the revised guideline which was published subsequently [5], highlight the importance of packed cell volume (PCV) and haemoglobin levels when deciding the quantity and the type of fluid replacement. Although all three guidelines consider third space fluid accumulation, such as, pleural effusions and ascites, in the diagnosis of DSS, they do not seem to emphasize their importance in management [3-5].

The commonly used fluids in the management of dengue fever include oral rehydration solutions (containing solutes), intravenous solutions such as crystalloids (isotonic fluid replacement) and colloids (plasma equivalents) [3-5]. The WHO guidelines in 1997 and 1999 recommend oral fluids and electrolyte therapy during the febrile phase and in patients with excessive sweating, vomiting and diarrhea, and those with evidence of plasma leakage as diagnosed by a rise in haemoglobin and haematocrit. The guidelines recommend fluid to be replaced initially with isotonic crystalloids such as 0.9% normal saline and Ringers lactate solution, which have equivalent composition to plasma fluid [3-5]. However, in cases with severe shock who do not respond to initial isotonic crystalloids resuscitation need to be treated with colloid solutions such as plasma or plasma substitutes such as Dextran 40 (10% dextran of medium related molecular mass in normal saline) or 5% albumin [3,4].

The recent outbreak of dengue in Sri Lanka accounted for more that 35000 cases and nearly 350 deaths (Epid Unit Colombo; Unpublished data). Many deaths were thought to be due to inappropriate fluid management leading to fluid overload (WHO expert statement, Colombo, Sri Lanka, Epid Unit Colombo; Unpublished data), in addition to myocarditis related cardiac failure and acute hepatic failure. We highlight three patients who were admitted to our unit in various stages of dengue infection and developed DSS. Although they were managed according to WHO guidelines [4] for fluid management, they later developed recovery phase pulmonary oedema resulting in extended morbidity in two patients and death in one. We feel that these cases highlight the importance of third space fluid loss when replacing fluids in DSS, so that recovery phase fluid overload and pulmonary oedema can be prevented.

## Case presentation

**Figure 1:**
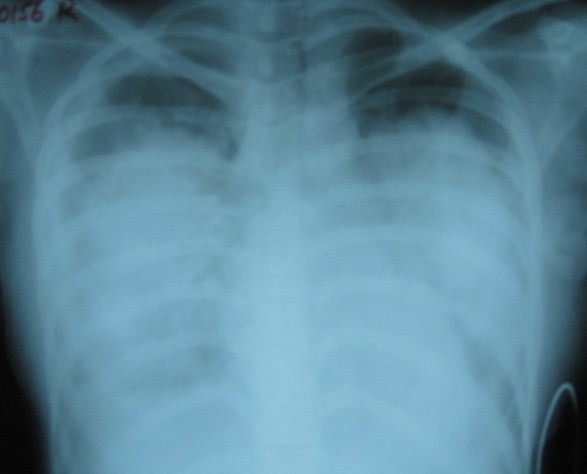
**Recovery phase pulmonary oedema with pleural effusion**.

**Figure 2:**
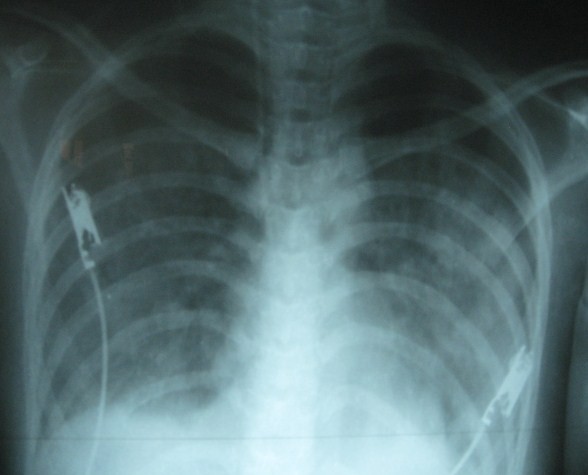
**Gradual improvement with CPAP mask ventilation**.

A 19-year-old girl with a body weight of 48 Kg, who presented with fever for 5 days, vomiting, headache, body aches and flushing developed dramatic reduction in blood pressures from 100/70 mmHg to 80/40 in the supine position, absent peripheral pulses and cold, clammy extremities on day 6 of the illlness. She developed no bleeding manifestations during her illness. Although she had an average Hb of 12.5 g/dl and a haematocrit of 42.3% before the development of DSS, at the time of development of DSS, she had a haematocrit of 55% with a Hb of 16 g/dl. She received 1.5 L of Ringers Lactate solution and 1 L of Normal saline over the 48 hours prior to developing DSS because she had intermittent vomiting during this period and she refused taking oral fluids and continued to vomit "all what she was consuming" on clinical assessment she had varying degrees of dehydration. Although such fluid replacement is not recommended in the WHO guidelines in the management of dengue fever we were compelled to introduce them purely on clinical grounds and on haemoglobin and haematocrit values. This was in addition to 1.5 L of oral fluid (consisting mainly of solutes; conjee and soup) over the same period "which she claimed to have vomited". Despite above management, she went into DSS very acutely and at the time of development of DSS it was noted that she had moderate ascites and a small right sided pleural effusion. She was then resuscitated primarily with 3 intermittent boluses (10-20 ml/Kg/hr) [1^st ^bolus (20 ml/Kg/Hr) over ~ 20 min (~320 ml), 2^nd ^bolus (10 ml/Kg/Hr) over ~ 20 min (~160 ml) and the 3^rd ^bolus (10 ml/Kg/Hr) over ~ 15 min (~120 ml); total of ~ 600 ml] and at a rate of ~ 2-4 ml/Kg/Hr in between boluses (~400 ml) of intravenous crystalloids amounting to 1000 ml over two hours and then with colloids (4 units of fresh frozen plasma ~100 ml × 4 = ~400 ml) followed by 5 ml/Kg/Hr for ~ 4 hours (~960 ml) according to 1999 WHO guidelines. We did not continue crystalloids in the same infusion rates for one hour as recommended by the WHO guidelines, and instead managed her with shorter duration boluses (15-20 min) due to fairly rapid accumulation of fluid in the third space. For the same reasons colloid solutions were instituted, as suggested in previous studies [6], when her haematocrit was increasing or was >50% in between fluid resuscitations. Her haematocrit level was repeatedly monitored during the 6 hours of resuscitation, and fluid management was strictly based on haematocrit levels. Her serum electrolytes, serum calcium, serum albumin and sugar levels were within normal limits throughout her illness, especially at the time she went into acute shock syndrome. Arterial blood gas analysis was not carried out during the acute stage (due to practical reasons; no peripheral arteries could be palpated, and more attention was given for resuscitation). She had a minimum urine output of 8 ml/hr for two hours during the period of DSS, but output improved thereafter, and she had no biochemical evidence of renal failure. She became mildly confused and drowsy with hepatic transaminases rising to very high levels (highest ALT- 1250 iu/L and highest AST- 4800 iu/L) with an INR of 1.8. She was given intravenous N-acetylcysteine [7], intravenous vitamin K and the liver failure regime (lactulose, metronidazole). Although she recovered from DSS within six hours of resuscitation, she developed progressive shortness of breath at rest, and this was observed within the next two hours. Her capillary oxygen saturation fell to 80 mmHg while on 60% O_2 _by face mask. Her chest x-ray showed severe pulmonary oedema with a right-sided pleural effusion (Figure 1). She had no evidence of myocarditis or other structural cardiac disease by ECHO cardiography, and had 60% ejection fraction at the time she developed pulmonary oedema. Her CVP was 20 cmH_2_O. She was commenced on continuous positive airway pressure (CPAP) mask ventilation with 100% O_2 _combined with intravenous frusemide, and showed gradual recovery over 72 hours (figure 2). Retrospective calculation reconfirmed that the volume of fluid she had received during the DSS phases of management were at volumes (in both oral and iv fluids) recommended by the WHO guidelines [4].

The second patient, was a 26 year old mother of two children with a body weight of 62 Kg who was admitted to hospital with profound DSS, together with moderate ascites and bilateral pleural effusions. Her systolic blood pressure on admission was 70 mmHg where the diastolic BP could not be recorded. The haemoglobin on admission was 16 g/dl with a haematocrit of 58% and the platelet count was 22,000/mm3 (these results were only available after an hour of commencing resuscitation). There was no evidence of bleeding. She had not been treated with intravenous fluids before admission, but was consuming oral fluids as advised by her general practitioner (the exact volumes she had consumed were not available as she had not maintained a fluid balance chart). Her ECG showed sinus tachycardia and widespraed T wave inversions, suggestive of myocarditis. However, she had a 55% ejection fraction by ECHO cardiography with no demonstrable underlying cardiac pathology. She was managed with two intermittent boluses (first bolus 20 ml/Kg/Hr over ~20 min amounting to ~400 ml and the other bolus 20 ml/Kg/Hr over another ~15 min amounting to ~300 ml) of intravenous normal saline (her body weight was assumed as 60 kg, as her body weight could not be measured as she was in profound shock on admission) and the total amount of crystalloids she had received was nearly 900 ml (Previous 700 ml + maintanence of 200 ml) during the first two hours (total volume give was and colloids [6 units of FFP (3 × ~100 ml + 3 × ~ 150 ml) amounting to ~750 ml)] over one hour. Fluid administration was titrated based on the haematocrit values. Here again short duration fluid boluses and colloids were given for the same reasons as the first patient. Although her blood pressures improved to 100/80 mm/Hg twice during resuscitation (After each bolus of crystalloids combined with colloids), this was not sustained. While being resuscitated she developed respiratory distress with evidence of pulmonary oedema in addition to bilateral pleural effusions and required intubation and ventilation. At the time she was transferred to the ICU for ventilation her CVP was 22 cm H_2_O and gradually rose to 26 cm H_2_O when she died 6 hours after admission to hospital. Her serum electrolytes were within normal limits, but blood gas analysis, carried out 2 hours after admission to the ICU, showed a mild metabolic acidosis. Her blood glucose remained normal but liver functions showed an AST of 5000 iu/L and ALT of 1600 iu/L. This patient was not commenced on N-acetylcysteine or the liver failure regime as she succumbed to her illness within 6 hours of admission and the liver function test results were available only after her death. The urine out put was only 40 ml during the 6 hours hospital stay, but the serum creatinine and blood urea were normal. The serum calcium and cholesterol levels could not be carried out in this patient. The post mortem showed large amounts of blood stained ascites and pleural effusions, congestion and widespread petecheal haemorrhages in all internal organs.

The third patient was a 32 year old female with a body weight of 58 Kg who was admitted on the 4^th ^day of her illness. She had not received intravenous fluids prior to admission and had a systolic BP of 90 mmHg and a diastolic BP of 80 mHg on admission, with a small right sided pleural effusion and mild ascites. On admission, her Hb was 17 g/dl and the PCV was 55, and the platelet count was 15,000 mm3. She was initially resuscitated with intravenous 0.9% saline 5-10 ml/Kg/Hr for 4 hours in order to maintain the blood pressure and she was advised to continue oral solutes thereafter. She maintained good urine out put of nearly 40-50 ml/Hr and had consumed nearly 1 L of oral solutes for the next 24 hours. However, 24 hours after admission, she developed profound shock with un-recordable systolic BP and absent peripheral pulses. Her PCV at this stage was 55 and she was intermittently resuscitated with three boluses of Ringers lactate (~300 ml) and normal saline solution [1^st ^bolus (20 ml/Kg/Hr) over ~ 15 min (~300 ml), 2^nd ^bolus (10 ml/Kg/Hr) over ~ 15 min (~150 ml) and the 3^rd ^bolus (20 ml/Kg/Hr) over ~ 20 min (~350 ml); total of ~ 600 ml] and at a rate of ~ 2-4 ml/Kg/Hr in between boluses (~400 ml) during the two hours of resuscitation (each bolus of fluid was given over 15-20 min durations and it was tapered to maintenance rates (5 ml/Kg/Hr or less) in between the boluses [the total amount of fluid that had been administered during this period was nearly 1200 ml], followed by 4 units of FFP (~100 ml × 1 + ~150 ml × 3 = ~550 ml) and 4 units of platelets (~75 ml × 4 = ~300 ml) (total of ~850 ml). Fluid resuscitation in this patient was also based on her haematocrit values. Her blood pressure improved after fluid resuscitation. During the two hours of shock stage she had urine out put of 50 ml and recovered thereafter. However, she rapidly developed tense ascites, and subsequently developed severe recovery phase pulmonary oedema and was treated with judicious amounts of intravenous frusemide, taking care of her blood pressures. She never had bleeding during the illness and her Hb and haematocrit dropped to 13 g/dl and 43% respectively after resuscitation. Her blood glucose, serum electrolytes and renal function remained normal throughout the illness, and AST and ALT levels were 1960 iu/L and 420 iu/L respectively. Although her blood gas analysis showed mild metabolic acidosis at the time she was sent to ICU for ventilation, it gradually normalized on subsequent monitoring. Her cardiac ejection fraction was 60% and had no structural cardiac illness by ECHO cardiography. Her CVP on admission to ICU was 18 cm H_2_O. Her serum Ca and cholesterol levels were not assessed. She was managed with CPAP ventilation similar to the first patient.

All these patients had continuous monitoring for vital signs, urine output and, when appropriate, haematocrit values. Full blood counts were carried out either twice daily or at an increased frequency when appropriate. Liver function tests were carried out at the onset and at the time of shock, and then as required for during follow up. Blood gases were done in the first and third patients at the time of the shock and when required thereafter. Serum calcium levels were measured only in the first patient and serum cholesterol levels were not assessed. Patients were advised to avoid water and were encouraged to take fluid containing electrolytes only. However, we have no details as to whether the second patient consumed water in excess amounts when she was at home.

## Conclusions

Two of our three patients had similar clinical and haematological parameters when they developed DSS. However the patient who died had no haematological investigations available at the time of presentation with profound DSS. These three patients were managed according to the WHO guidelines. We carried out most of the appropriate investigations and used the fluid management algorhythms. Although we used both crystalloids and colloids in the resuscitation of all three patients, crystalloids were prioritized over colloids even after the detection of third space fluid accumulation. However, although the WHO guidelines recommend fluid boluses at increased rates for one hour, we were compelled to use shorter duration fluid boluses and intermittent colloids as the patients were rapidly developing third space fluid accumulation in association with an increase in haematocrit, suggesting continued fluid leakage. Such continued fluid leakage could be due to uncorrected hypoglycaemia, acidosis, low serum calcium, lowering of serum albumin or due to administration of either iv or oral hypotonic fluids in excess. The patient who died had not received intravenous fluids prior to admission, but she had been consuming extra fluids at home, which is currently recommended for home based management of DF. However the volume and the composition of fluid consumed had not been documented by the patient. The other two patients did not receive iv or extra oral fluids prior to or after admission. In all three patients the blood sugar level remained normal. In the first patient, the corrected serum calcium remained normal and albumin level was low normal. In patients 1 and 3 there was only a mild metabolic acidosis. In the second patient, some of these investigation results were either not carried out or not available until very close to her time of death. During the convalescence, all three patients developed pulmonary oedema and reduced arterial oxygen saturation requiring intensive care treatment with ventilation. Observing the clinical features, the management and the outcome of these three patients, we hypothesize that resuscitation of patients who already have third space fluid accumulation at the time of development of severe DSS giving priority to colloids rather than to crystalloids would prevent the development of recovery phase pulmonary oedema.

Infections are a major cause of circulatory collapse in children or adults [8]. However the controversy over the choice of resuscitation solution, whether it is crystalloids or colloids is yet to be resolved [8,9]. Although there are several studies that has been performed in order to address this issue in both children and adults, most these studies seem to depend on parameters such as blood pressure, pulse rate, capillary filling time, pulse pressure, urine output haemoglobin and pack red cell volume in the diagnosis and evaluation of treatment outcomes [10,11]. They do not consider the presence of third space fluid loss as an important determinant in order to select the resuscitation fluids. Although the pathophysiology of shock syndrome due to most infections is similar [9], the shock syndrome in DF is transient and reverses rapidly [3-5]. Therefore, patients who developed third space fluid loss during shock stage are very likely to be at risk of rapid circulatory overload during the recovery phase. There is one randomized controlled trial that highlights the advantage of colloids over crystalloids in the management of DSS in children [11]

All these patients were assessed and resuscitated according to WHO guidelines for the management of dengue fever [4]. As highlighted in the WHO guidelines, there is no doubt that haematological parameters such as Hb and haematocrit, and assessment for evidence for third space fluid loss such as the presence of ascites and pleural effusions are very important in diagnosing early fluid leakage and subsequent intravascular volume loss. However the guidelines do not pay much attention to the presence of third space fluid accumulation when selecting appropriate intravenous fluids for resuscitation [3-5]. We wonder whether resuscitation of intravascular volume in patients who develop DSS in the presence of ascites and pleural effusions, giving priority to colloid boluses instead of crystalloid boluses would prevent fluid overload related morbidity and mortality in the recovery phase. This is because, crystalloids (normal saline) increases the extracellular fluid compartment 1:1 ratio with only 20% remaining with the intravascular space, whereas 5% albumin increases extracellular fluid more than twice and is distributed equally intravascularly and interstitially [12]. Therefore, volume to volume, two to three times as much crystalloids need to be used as colloid for the same haemodynamic effect. Furthermore, a higher incidence of pulmonary oedema has been reported in patients with hypovolemic and septic shock receiving saline, which was attributed to a fall in colloid osmotic pressure [13]. Therefore, replacing crystalloids with colloid solutions, such as FFP, may reduce recovery phase pulmonary oedema by improving osmotic pressure resulting in minimizing leakage of fluid to the extravascular compartment. Furthermore, such improvement in oncotic pressure would also help in the gradual re-absorption of ascites and pleural effusions. This may also reduce requirements for intravenous crystalloids in the management of DSS.

A previous double blind randomized clinical trial conducted in children with moderately severe DSS has shown that there was no significant difference in the development of new bleeding manifestations, clinical fluid overload, objective measures of the overall severity of vascular leakage (right pleural effusion, ascites), or the use of furosemide whether they were initially resuscitated with crystalloids or colloids [6]. However, in this study, the presence of third space fluid accumulation at the time of recruitment to the study is not mentioned. Furthermore, the study does not randomize patients with severe dengue shock syndrome to treatment with crystalloids because of concerns about the potential development of critical fluid overload without access to advanced respiratory support. However, more patients with severe shock at presentation had required rescue colloids than did patients with moderately severe shock. The article also highlights the finding that the effect of colloids in preventing fluid leakage lasts only a few hours. However it concludes that for those with severe shock, the situation is less clear-cut and that clinicians must continue to rely on personal experience, familiarity with particular products, local availability, and cost. Minor advantages in initial recovery were shown with starch, and significantly more adverse reactions were associated with dextran, so that if the use of a colloid is considered necessary, starch may be the preferred option.

In conclusion, we recommend that our observation, that resuscitation of patients with DSS who already have third space fluid accumulation with crystalloid boluses on a priority basis may contribute to recovery phase pulmonary oedema, needs to be further tested by randomized control trials recruiting patients with severe DSS who have already developed third space fluid accumulation. Use of colloids such as FFP could be investigated in such trials, keeping in mind possible transfusion reactions with FFP and reactions to artificial colloids while they are being administered.

## Consent

Written informed consent was obtained from the patients (Patient number 1 and 3) and the husband of patient number 2 for publication of this case report and the accompanying images. A copy of the written consent is available for review by the Editor-in-Chief of this journal.

## Competing interests

The authors declare that they have no competing interests.

## Authors' contributions

RP, LEW*, WOMS* and HJdeS: Management of all three patients, critical analysis of management data, writing up the manuscript*, revising with new data*. All authors read the final paper and agreed upon its contents.

## Pre-publication history

The pre-publication history for this paper can be accessed here:

http://www.biomedcentral.com/1471-2334/11/52/prepub
